# First Documented Transmission of *Trypanosoma cruzi* Infection through Blood Transfusion in a Child with Sickle-Cell Disease in Belgium

**DOI:** 10.1371/journal.pntd.0003986

**Published:** 2015-10-15

**Authors:** Sophie Blumental, Micheline Lambermont, Catherine Heijmans, Marie-Pierre Rodenbach, Hanane El Kenz, Danièle Sondag, Emmanuel Bottieau, Carine Truyens

**Affiliations:** 1 Pediatric Infectious Disease Unit, Hôpital Universitaire des Enfants Reine Fabiola, Brussels, Belgium; 2 Service du Sang, Belgian Red Cross, Brussels, Belgium; 3 Hemato-oncology Department, Hôpital Universitaire des Enfants Reine Fabiola, Brussels, Belgium; 4 Department of Clinical Sciences, Institute of Tropical Medicine, Antwerp, Belgium; 5 Laboratory of Parasitology, Faculty of Medicine, Université Libre de Bruxelles, Brussels, Belgium; Universidad Autónoma de Yucatán, MEXICO

## Introduction

Chagas disease is a parasitic infection due to *Trypanosoma cruzi* that is endemic in Latin America (LA), where *Triatominae* vectors are present. The parasite, multiplying inside various cell types, disseminates into its vertebrate host as blood trypomastigotes, and can be transmitted through blood transfusion as well as by organ transplantation or congenitally [1]. Due to important migration of chronically infected people from LA, Chagas disease is now encountered in non-endemic countries as well [2,3]. Although the actual prevalence of Chagas disease in most European countries remains poorly quantified, a recent systematic review conducted in those countries that welcome the most immigrants from LA estimated this prevalence at 4.2% on average, plausibly corresponding to at least 100,000 *T*. *cruzi*-infected individuals in Europe [3–5]. Chronically infected people, though harboring very low parasite burden, remain with a lifelong risk of not only developing severe cardiac or digestive complications but also of causing disease transmission in host countries. *T*. *cruzi* transmission through transplantation, blood transfusion, and maternal-fetal transmission is increasingly reported outside of LA, including in Europe—particularly in southern countries like Spain and Italy, where immigration from LA is the highest [3,5–16].

So far, a systematic *T*. *cruzi* screening of transfusion-dedicated blood components obtained from at-risk donors has not been organized in all European countries, particularly in northern Europe but with some exceptions [5]. In Belgium, donors born in an endemic country or with a history of travel to endemic countries were, until recently, only excluded from donation during the six months following the last “at-risk” trip [5]. From Fall 2013 onward, following recommendations of the Belgian Federal Agency for Medicines and Health Products (FAMHP), the Belgian blood bank services decided to perform a serological screening for *T*. *cruzi* in all those donors who were previously only temporary deferred, i.e., in donors born in an endemic country or with a history of residence or travel to endemic countries but regardless of the elapsed time between the stay and blood donation.

We report here the first case in Belgium of Chagas infection transmitted by red blood cell (RBC)-transfusion occurring in a child suffering from sickle-cell disease. The child’s infection was detected soon after a systematic screening was implemented in our country. We thereby highlight the usefulness of *T*. *cruzi* systematic screening among at-risk donors everywhere in Europe—even in countries with rather low Latin American immigration, like Belgium—and emphasize the importance of increasing clinicians’ awareness about Chagas disease in patients receiving recurrent blood transfusions.

## Case Presentation

**Fig 1:**
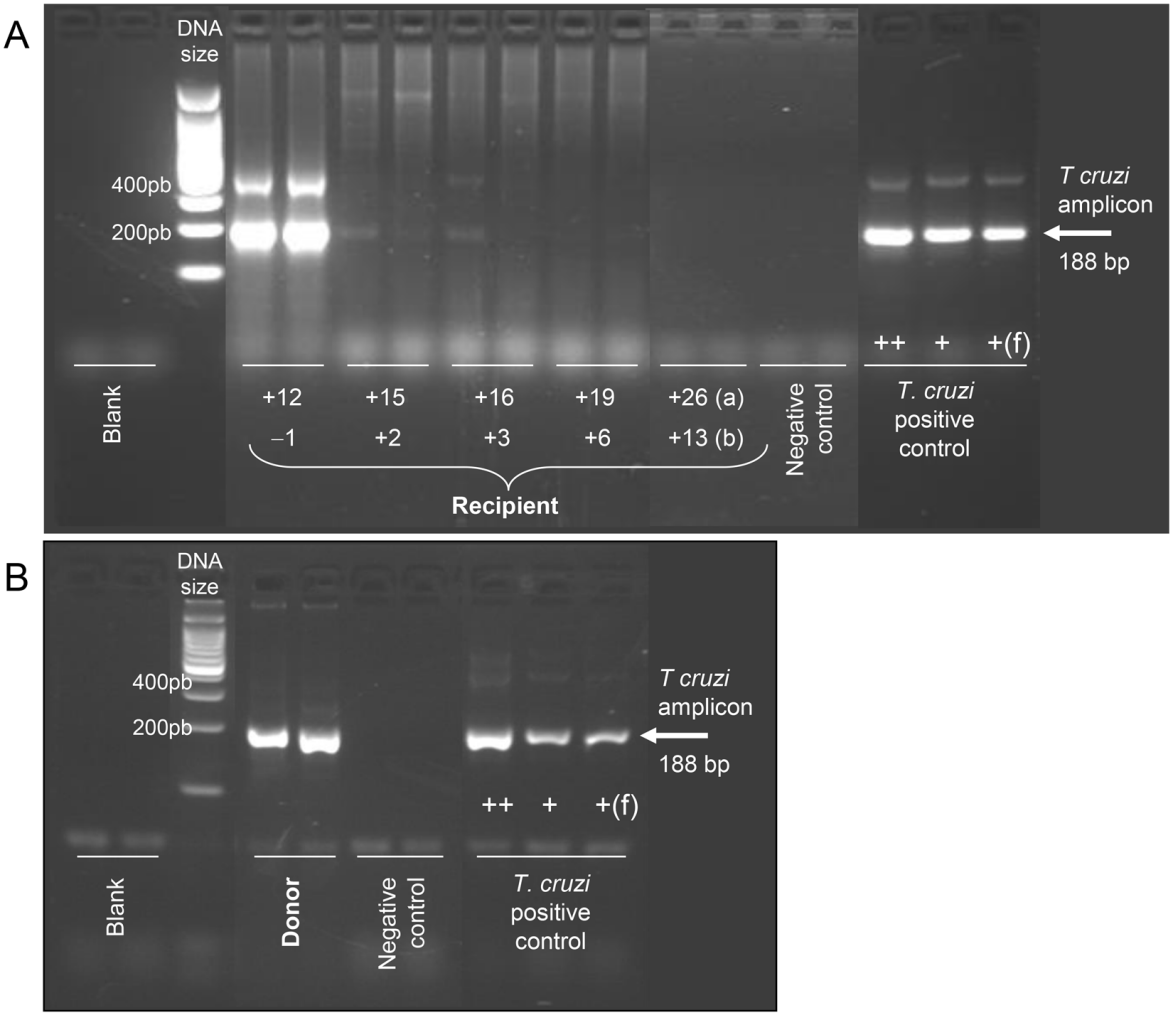
*T*. *cruzi* PCR results in the contaminated recipient (A) and the infected donor (B). In the recipient, PCR has been performed at 12, 15, 16, 19, and 26 months after *T*. *cruzi* contamination occurred (A), which corresponds to one month before and two, three, six, and 13 months after initiation of treatment with benznidazole, respectively (B). PCR was performed in duplicates on DNA extracted from guanidine-preserved blood samples with TcZ1-TcZ2 primers, giving an amplicon of 188 basepairs (bp), indicated by the arrow [20]. Additional bands around 400 and 600 bp are sometimes visible. They originate from tandem repetitions of the target sequence in *T*. *cruzi* DNA. Parasite purified DNA from 10, 1, and 0.1 parasites/mL was used as positive control. Blood sample from an uninfected individual was used as negative control.

The patient was a 7-year-old boy, born in Belgium from two parents of Burundi origin, who suffered from sickle-cell disease that was diagnosed during the neonatal period. He was undergoing manual recurrent partial exchange transfusion (roughly once a month since the age of four) in combination with medical treatment (hydroxyurea). Despite suitable therapy, he presented with some complications that mainly consisted of neurovascular malformations (Moyamoya disease). A look-back procedure, launched soon after an infected donor was detected through the new screening program implemented in Belgium in September 2013, revealed that this child had received contaminated RBCs eight months earlier. Subsequent serological investigations showed that the child’s serum was reactive in the three tests used in the national reference laboratory (Table 1). The diagnosis of *T*. *cruzi* infection was finally confirmed in another blood sample, collected three weeks later, by the demonstration of *T*. *cruzi* DNA by PCR (Fig 1 and Table 1). Since a pre-transfusion sample tested retrospectively was proven to be non-reactive against *T*. *cruzi*, the imputability of the Chagas contamination to the incriminated blood donation could be definitively established in this young patient who had never travelled to South America. No symptom suggestive of acute Chagas disease could be retrospectively noticed in the period of time elapsing from blood donation to infection diagnosis. An antiparasitic treatment (benznidazole), obtained through the donation program from the World Health Organization in Geneva, was administered at a dosage of 5 mg/kg twice daily, for a duration of eight weeks. As no formal information was available about interactions between benznidazole and hydroxyurea—two drugs that are potentially hematotoxic [17,18]—the latter drug was suspended during the first two weeks of antiparasitic therapy but was resumed later without any inconvenience. Tolerance was markedly good, with no clinical or biological adverse reaction observed throughout the whole treatment. Only the neurosurgery that was planned for his significant neurovascular lesions had to be delayed during the medical treatment. Unfortunately, a cerebrovascular ischemic event, as commonly observed in Moyamoya disease, occurred in the meanwhile but was of short duration and recovery was spontaneously complete. *T*. *cruzi* DNA remained detectable by PCR in the patient’s blood two and three months after the antiparasitic treatment was started, but intensity of amplicon bands was markedly decreased compared to that observed at the time of infection diagnosis (Fig 1A). The PCR became negative three months after completion of therapy; this result, however, does not guarantee a complete cure of the child since PCR might be falsely negative in the case of very low parasite burden. Extended follow-up with repeated testing is therefore planned to sustain long-term resolution of infection and absence of chronic complications. At present, more than two years after the contaminated transfusion occurred and one year after treatment completion, the child remained asymptomatic, and parasite DNA was undetectable in blood samples by PCR (as assessed in June 2015). He was still regularly receiving blood transfusion/manual partial exchanges, which are the only option in sickle-cell patients waiting for curative bone marrow transplant.

**Table 1:** Evolution of *T*. *cruzi* serology and PCR testing in the child contaminated by RBC transfusion.

Time of sampling		*T*. *cruzi* specific antibodies^a^			*T*. *cruzi* PCR^b^
*Months after T*. *cruzi contamination*	*Months after treatment initiation* ^c^	Bioelisa Chagas^d^ Result (ratio)	In house ELISA^e^ Result (index)	Vircell IFA^f^ Result (titer)	Result
**˗** 3 ½		neg (0.26)	neg (0)	neg (<40)	nd^g^
+ 8 ½		pos (3.44)	pos (62)	pos (1,280)	nd
+ 12	˗ 1	nd	nd	nd	pos ++
+ 15	+ 2	pos (5.97)	pos (68)	pos (2,560)	weakly pos
+ 16	+ 3	nd	nd	nd	weakly pos
+ 19	+ 6	pos (1.74)	pos (57)	pos (640)	neg
+ 26	+ 13	pos (2.71)	pos (60)	pos (640)	neg

^a)^ These tests were performed at the Belgian reference center. According to WHO recommendations [19], chronic infection with *T*. *cruzi* needs two positive serological assays, using different techniques, to be diagnosed.

^b)^ PCR performed as described in Fig 1

^c)^ Benznidazole has been given for a duration of two months

^d)^ Biokit S.A., Barcelona, Spain—Positive result when ratio > 1 (cf. manufacturer instructions)

^e)^ ELISA based on soluble extract of *T*. *cruzi* trypomastigotes—Positive result if index > 20 (positive control index = 100, negative control index = 0)

^f)^ Chagas IFA IgG+IgM, Vircell, Granada, Spain—Positive result when titer ≥ 40 (cf. manufacturer)

^g)^ nd: not determined

The contaminating donor was a 47-year-old male born in an endemic rural area near Santa Vitoria in Brazil, where he lived until the age of 23 before moving to São Paulo and then to Belgium when he was about forty. Shortly after implementation of *T*. *cruzi* blood screening in Belgium, he was found to be positive with four different serological assays that all remained reactive in another sample obtained three months later (Table 2). A positive *T cruzi* PCR confirmed the possible disease activity and infectivity (Fig 1B). The donor was fully asymptomatic, had no clinical sign of chronic Chagas disease, and did not report any history of Chagas disease in his family, though he was aware of the risk of infection because of insecticide spraying performed annually in his home in Santa Vitoria. Despite repeated requests by the blood bank services that he see an infectious disease specialist, the infected donor has been unfortunately lost to follow-up. The look-back procedure revealed that two other patients had received blood products from the same infected donor. The first one was a 43-year-old woman who received RBC within the care of extensive burns. She died one month later from the underlying pathology, and no further evaluation of the Chagas status could be performed. The other recipient was a 40-year-old man with HIV infection, a herpes virus 8-associated lymphoma, and Kaposi’s sarcoma. He received a pool of platelet concentrates prepared from six leuko-reduced whole blood samples (one of which was from the infected donor) and was treated for pathogen reduction with amotosalen and UVA (INTERCEPT, Cerus Europe BV, Netherlands). Such treatment has been shown to efficiently inactivate *T*. *cruzi* [21], which is important because, without treatment, parasites remain detectable at low level in platelet concentrates [22]. No evidence of *T*. *cruzi* infection was found in this patient.

**Table 2:** Evolution of *T*. *cruzi* serology and PCR testing in the blood donor.

Time of sampling	*T*. *cruzi* specific antibodies				*T*. *cruzi* PCR^b^
*Months after diagnosis of T*. *cruzi infection*	CMIA^a^ Result (unit)	Bioelisa Chagas^d^ Result (ratio)	In house ELISA^e^ Result (index)	Vircell IFA^f^ Result (titer)	Result
0	pos (9.38)	pos (2.44)	pos (113)	pos (1280)	nd^g^
+ 3	pos (9.81)	pos (5.17)	pos (129)	pos (1280)	pos ++

^a)^ Chemoluminescent microparticle immunoassay, Architect Chagas Assay, Abbott Laboratories, Germany (Positive result when unit > 1). This test is used for blood donors screening at the blood transfusion center.

^b)^ to g) cf. Table 1

## Case Discussion

We report here the first well-documented case of *T*. *cruzi* transmission in Belgium that was retrospectively diagnosed thanks to the recent implementation of *T*. *cruzi* infection screening among blood donors and a subsequent look-back procedure in our country. This is also the first case, to our knowledge, of the administration of benznidazole treatment in a child chronically treated by hydroxyurea for sickle-cell disease.

In contrast to the situation in southern Europe, Chagas disease is often hardly considered or assessed in most northern European countries, where the immigration flow from LA is much more limited [13]. In Belgium, from Fall 2013 onward, the blood bank services decided to perform a broader serological screening for *T*. *cruzi* in all at-risk donors born in an endemic country who were previously only temporary deferred from donation (see definitions above). The screening procedure was similar to that which was implemented in several countries from North America and Europe that face large immigration from LA [5,11,23–26]. The risk of Chagas transmission after transfusion of blood products from an asymptomatic but *T*. *cruzi*-contaminated donor (as defined by positive serology or PCR) remains, however, poorly quantified [1]. According to a recent systematic review, only a few well-documented cases of transfusion-related contamination have been described in non-endemic countries such as the United States or Spain, where seroprevalence of *T*. *cruzi* infection among blood donors is relatively high [24]. In Spain, Piron et al. reported that 0.62% of at-risk donors (i.e., having resided in an endemic region, including Spanish travelers) were seropositive to *T*. *cruzi* [27]. Another study in France found a prevalence of positive *T*. *cruzi* serology of 0.01% amongst at-risk screened donors [23]. In addition, a Swiss study recently reported that about 15% of immigrants from LA diagnosed with Chagas disease had already donated blood previously and that another 25% expressed willingness to donate blood or an organ [28]. These observations well illustrate the “ongoing” risk of Chagas transmission in all European countries that harbor exposed communities (even those that are limited in size) if blood or organ donations remain unscreened. Whereas the efficacy and cost effectiveness of such a screening procedure remains to be thoroughly assessed in western Europe, our case highlights its importance, at least in clinical care. A more proactive approach is also particularly relevant in an era when using aggressive immunosuppressive therapies and subsequently resorting to blood transfusions is increasing. Of note, in our case, *T*. *cruzi* transmission occurred through transfusion of red blood cell preparation, whereas most other reports have primarily incriminated platelet concentrates [1,11]. Additional control measures, such as pathogen reduction systems applicable to all blood components, have to be further explored in order to reduce the continuous threats from Chagas and other new emerging transmissible agents [29,30].

The administration of benznidazole therapy to a child chronically treated with hydroxyurea for sickle-cell disease is another rather unique feature of our case report. After demonstrating that *T*. *cruzi* infection in the child was still ongoing eight months after transfusion (suggesting that he was evolving to an early chronic stage), a treatment with an antiparasitic drug was required without delay. This was justified by the fact that, firstly, the success of treatment with benznidazole or nifurtimox—the two currently available antiparasitic drugs that are active against *T*. *cruzi*—is substantially higher if it is given during recent/acute infection rather than during the chronic phase. In endemic areas, the success rate of etiological treatment is globally estimated at about 80% when initiated during acute phase (within four months after infection), 50%–60% during recent chronic phase (after four months of infection in children 0–14 years), or <50% during late chronic phase in any case ≥15 years of age [31,32]. Secondly, successful treatment would limit the risk for this child to develop long-term severe cardiac or digestive complications (estimated at 30%–40% in non-treated individuals [1]). Finally, the patient was eligible for bone marrow transplantation to cure his sickle-cell disease, with a 17–40% risk of serious Chagas reactivation in case of persistent active infection [33]. Risk of post-transplantation reactivation varies according to the type of transplant and has not been prospectively quantified in the context of hematopoietic stem cell transplantation [34]. However, life-threatening Chagas reactivation with involvement of the central nervous system has been reported in such patients on several occasions, even in Europe [33,35]. Although the efficacy of both antiparasitic drugs against Chagas disease is reportedly similar, the drug currently provided by the WHO in Europe, benznidazole, is preferred over nifurtimox because of very poor tolerance to the latter. In a recent observational prospective study including 81 Bolivian adults treated with nifurtimox in Switzerland, almost all patients experienced adverse events (7.4% defined as serious), which caused treatment discontinuation in 50% of the cohort [36]. Benznidazole is usually better tolerated (with a treatment completion rate of about 85%) although side effects are commonly described and include hypersensitivity-related rash (sometimes with angioedema), gastro-intestinal intolerance, peripheral neuropathy (at the end of the treatment), or myelosuppression [17,37]. Despite an extensive literature search, no information could be retrieved on potential risks of combining benznidazole and hydroxyurea—both drugs that are potentially associated with hematotoxicity. We therefore opted for a sequential use of these two drugs when starting therapy. The child did not experience any adverse drug reaction during the period of benznidazole monotherapy nor thereafter, when it was combined with hydroxyurea after reintroduction of this latter drug. The evolution was marked by a transient ischemic attack for which a causative link with the short hydoxyurea interruption could not be established.

In conclusion, the case reported here illustrates once again how complex the management of *T*. *cruzi* infection can be. Even if rather infrequent, Chagas infection has to be considered, even in European countries that host a lower population from LA, such as Belgium. Substantial efforts are still necessary to increase clinicians’ awareness about the disease manifestations and the risk of secondary transmission, as well as to improve the recognition of latent infection in at-risk blood and organ donors and pregnant women.

**Key Learning Points**

- Chagas disease, due to the protozoon *Trypanosoma cruzi*, is still an important cause of morbidity and mortality in some regions of Latin America, where it is endemic.
- Chagas disease is increasingly encountered in Europe, North America, and other non-endemic countries due to immigration of asymptomatic and undiagnosed chronically infected people from Latin America. In these countries, transmission may occur through blood transfusion, organ transplantation, and congenitally.
- In some European countries where immigration of Latin American people is rather limited, like Belgium, most physicians are not aware of the risk of *T*. *cruzi* secondary transmission. Among other measures, it is important to set up a systematic screening of at-risk blood donors for Chagas disease, even in such countries.
- Efficient pathogen reduction systems applicable to all blood components, and not only to platelet concentrates and plasma, should be explored to reduce the threat of contracting Chagas and other emerging blood transmissible agents.
- Treatment of Chagas disease with benznidazole was administered uneventfully to the patient we reported here, who was a child also treated with hydroxyurea for sickle-cell disease.

## References

[1] Rassi A Jr., Rassi A, Marin-Neto JA. Chagas disease. Lancet. 2010 4 17;375(9723):1388–402. 10.1016/S0140-6736(10)60061-X 20399979

[2] Schmunis GA, Yadon ZE. Chagas disease: a Latin American health problem becoming a world health problem. Acta Trop. 2010 7;115(1–2):14–21. 10.1016/j.actatropica.2009.11.003 19932071

[3] Basile L, Jansa JM, Carlier Y, Salamanca DD, Angheben A, Bartoloni A, et al Chagas disease in European countries: the challenge of a surveillance system. Euro Surveill. 2011;16(37). 21944556

[4] Requena-Mendez A, Aldasoro E, de Lazzari E, Sicuri E, Brown M, Moore DA, et al Prevalence of Chagas disease in Latin-American migrants living in Europe: a systematic review and meta-analysis. PLoS Negl Trop Dis. 2015 2;9(2):e0003540 10.1371/journal.pntd.0003540 25680190 PMC4332678

[5] Requena-Mendez A, Albajar-Vinas P, Angheben A, Chiodini P, Gascon J, Munoz J. Health policies to control Chagas disease transmission in European countries. PLoS Negl Trop Dis. 2014 10;8(10):e3245 10.1371/journal.pntd.0003245 25357193 PMC4214631

[6] Villalba R, Fornes G, Alvarez MA, Roman J, Rubio V, Fernandez M, et al Acute Chagas' disease in a recipient of a bone marrow transplant in Spain: case report. Clin Infect Dis. 1992 2;14(2):594–5. 1554849 10.1093/clinids/14.2.594

[7] Lescure FX, Canestri A, Melliez H, Jaureguiberry S, Develoux M, Dorent R, et al Chagas disease, France. Emerg Infect Dis. 2008 4;14(4):644–6. 10.3201/eid1404.070489 18394284 PMC2570909

[8] Buekens P, Almendares O, Carlier Y, Dumonteil E, Eberhard M, Gamboa-Leon R, et al Mother-to-child transmission of Chagas' disease in North America: why don't we do more? Matern Child Health J. 2008 5;12(3):283–6. 17602289 10.1007/s10995-007-0246-8

[9] Munoz J, Coll O, Juncosa T, Verges M, del Pino M, Fumado V, et al Prevalence and vertical transmission of Trypanosoma cruzi infection among pregnant Latin American women attending 2 maternity clinics in Barcelona, Spain. Clin Infect Dis. 2009 6 15;48(12):1736–40. 10.1086/599223 19438393

[10] Jackson Y, Myers C, Diana A, Marti HP, Wolff H, Chappuis F, et al Congenital transmission of Chagas disease in Latin American immigrants in Switzerland. Emerg Infect Dis. 2009 4;15(4):601–3. 10.3201/eid1504.080438 19331743 PMC2671437

[11] Perkins HA, Busch MP. Transfusion-associated infections: 50 years of relentless challenges and remarkable progress. Transfusion. 2010 10;50(10):2080–99. 10.1111/j.1537-2995.2010.02851.x 20738828

[12] Gascon J, Bern C, Pinazo MJ. Chagas disease in Spain, the United States and other non-endemic countries. Acta Trop. 2010 7;115(1–2):22–7. 10.1016/j.actatropica.2009.07.019 19646412

[13] Carlier Y. Globalization of Chagas disease (American trypanosomiasis): the situation in Europe and Belgium. Bull Mem Acad R Med Belg. 2011;166(10–12):347–55. 23082500

[14] Hotez PJ, Dumonteil E, Betancourt CM, Bottazzi ME, Tapia-Conyer R, Meymandi S, et al An unfolding tragedy of Chagas disease in North America. PLoS Negl Trop Dis. 2013;7(10):e2300 10.1371/journal.pntd.0002300 24205411 PMC3814410

[15] Fores R, Sanjuan I, Portero F, Ruiz E, Regidor C, Lopez-Velez R, et al Chagas disease in a recipient of cord blood transplantation. Bone Marrow Transplant. 2007 1;39(2):127–8. 17213850 10.1038/sj.bmt.1705551

[16] Flores-Chavez M, Fernandez B, Puente S, Torres P, Rodriguez M, Monedero C, et al Transfusional chagas disease: parasitological and serological monitoring of an infected recipient and blood donor. Clin Infect Dis. 2008 3 1;46(5):e44–e47. 10.1086/527448 18257698

[17] Altcheh J, Moscatelli G, Moroni S, Garcia-Bournissen F, Freilij H. Adverse events after the use of benznidazole in infants and children with Chagas disease. Pediatrics. 2011 1;127(1):e212–e218. 10.1542/peds.2010-1172 21173000

[18] Ware RE, Aygun B. Advances in the use of hydroxyurea. Hematology Am Soc Hematol Educ Program. 2009;62–9. 10.1182/asheducation-2009.1.62 20008183

[19] World Health Organization (WHO). Control of Chagas disease: second report of the WHO expert committee. Geneva: WHO; 2002. Report No.: 905.

[20] Virreira M, Torrico F, Truyens C, Alonso-Vega C, Solano M, Carlier Y, et al Comparison of polymerase chain reaction methods for reliable and easy detection of congenital Trypanosoma cruzi infection. Am J Trop Med Hyg. 2003 5;68(5):574–82. 12812349 10.4269/ajtmh.2003.68.574

[21] Castro E, Girones N, Bueno JL, Carrion J, Lin L, Fresno M. The efficacy of photochemical treatment with amotosalen HCl and ultraviolet A (INTERCEPT) for inactivation of Trypanosoma cruzi in pooled buffy-coat platelets. Transfusion. 2007 3;47(3):434–41. 17319823 10.1111/j.1537-2995.2007.01133.x

[22] Cancino-Faure B, Fisa R, Riera C, Bula I, Girona-Llobera E, Jimenez-Marco T. Evidence of meaningful levels of Trypanosoma cruzi in platelet concentrates from seropositive blood donors. Transfusion. 2015 6;55(6):1249–55. Epub 2015 Feb 13. 10.1111/trf.12989 25683267

[23] El Ghouzzi MH, Boiret E, Wind F, Brochard C, Fittere S, Paris L, et al Testing blood donors for Chagas disease in the Paris area, France: first results after 18 months of screening. Transfusion. 2010 3;50(3):575–83. 10.1111/j.1537-2995.2009.02476.x 19906038

[24] Benjamin RJ, Stramer SL, Leiby DA, Dodd RY, Fearon M, Castro E. Trypanosoma cruzi infection in North America and Spain: evidence in support of transfusion transmission. Transfusion. 2012 9;52(9):1913–21. 10.1111/j.1537-2995.2011.03554.x 22321142

[25] Kitchen AD, Hewitt PE, Chiodini PL. The early implementation of Trypanosoma cruzi antibody screening of donors and donations within England: preempting a problem. Transfusion. 2012 9;52(9):1931–9. 10.1111/j.1537-2995.2012.03599.x 22414025

[26] O'Brien SF, Scalia V, Goldman M, Fan W, Yi QL, Dines IR, et al Selective testing for Trypanosoma cruzi: the first year after implementation at Canadian Blood Services. Transfusion. 2013 8;53(8):1706–13. 10.1111/j.1537-2995.2012.03950.x 23145895

[27] Piron M, Verges M, Munoz J, Casamitjana N, Sanz S, Maymo RM, et al Seroprevalence of Trypanosoma cruzi infection in at-risk blood donors in Catalonia (Spain). Transfusion. 2008 9;48(9):1862–8. 10.1111/j.1537-2995.2008.01789.x 18522707

[28] Jackson Y, Getaz L, Wolff H, Holst M, Mauris A, Tardin A, et al Prevalence, clinical staging and risk for blood-borne transmission of Chagas disease among Latin American migrants in Geneva, Switzerland. PLoS Negl Trop Dis. 2010;4(2):e592 10.1371/journal.pntd.0000592 20126397 PMC2814851

[29] Schmidt M, Geilenkeuser WJ, Sireis W, Seifried E, Hourfar K. Emerging Pathogens—How Safe is Blood? Transfus Med Hemother. 2014 2;41(1):10–7. 10.1159/000358017 24659943 PMC3949612

[30] Schubert P, Culibrk B, Karwal S, Serrano K, Levin E, Bu D, et al Whole blood treated with riboflavin and ultraviolet light: quality assessment of all blood components produced by the buffy coat method. Transfusion. Epub 2014 Oct 29. 10.1111/trf.12895 25355434

[31] Guedes PM, Silva GK, Gutierrez FR, Silva JS. Current status of Chagas disease chemotherapy. Expert Rev Anti Infect Ther. 2011 5;9(5):609–20. 10.1586/eri.11.31 21609270

[32] Fabbro DL, Streiger ML, Arias ED, Bizai ML, del Barco M, Amicone NA. Trypanocide treatment among adults with chronic Chagas disease living in Santa Fe city (Argentina), over a mean follow-up of 21 years: parasitological, serological and clinical evolution. Rev Soc Bras Med Trop. 2007 1;40(1):1–10. 17486245 10.1590/s0037-86822007000100001

[33] Altclas J, Sinagra A, Dictar M, Luna C, Veron MT, De Rissio AM, et al Chagas disease in bone marrow transplantation: an approach to preemptive therapy. Bone Marrow Transplant. 2005 7;36(2):123–9. 15908978 10.1038/sj.bmt.1705006

[34] Lattes R, Lasala MB. Chagas disease in the immunosuppressed patient. Clin Microbiol Infect. 2014 4;20(4):300–9. 10.1111/1469-0691.12585 24602129

[35] Altclas J, Salgueira C, Riarte A. Reactivation of Chagas disease after a bone marrow transplant. Blood Transfus. 2014 1;12 Suppl 1:s380 10.2450/2013.0009-13 23867177 PMC3934264

[36] Jackson Y, Alirol E, Getaz L, Wolff H, Combescure C, Chappuis F. Tolerance and safety of nifurtimox in patients with chronic chagas disease. Clin Infect Dis. 2010 11 15;51(10):e69–e75. 10.1086/656917 20932171

[37] Viotti R, Vigliano C, Lococo B, Alvarez MG, Petti M, Bertocchi G, et al Side effects of benznidazole as treatment in chronic Chagas disease: fears and realities. Expert Rev Anti Infect Ther. 2009 3;7(2):157–63. 10.1586/14787210.7.2.157 19254164

