# Correction: First Documented Transmission of *Trypanosoma cruzi* Infection through Blood Transfusion in a Child with Sickle-Cell Disease in Belgium

**DOI:** 10.1371/journal.pntd.0004665

**Published:** 2016-04-20

**Authors:** Sophie Blumental, Micheline Lambermont, Catherine Heijmans, Marie-Pierre Rodenbach, Hanane El Kenz, Danièle Sondag, Emmanuel Bottieau, Carine Truyens

The affiliation for the fifth author is incorrect. Hanane El Kenz is affiliated with Brugmann University Hospital Center and Queen Fabiola University Children Hospital Blood Bank Department, Brussels, Belgium.
